# Optical coherence tomography angiography in patients with focal epilepsy

**DOI:** 10.3389/fneur.2025.1529409

**Published:** 2025-01-29

**Authors:** Flora Rider, Alla Guekht, Alexander Shpak

**Affiliations:** ^1^Moscow Research and Clinical Center for Neuropsychiatry, Moscow, Russia; ^2^Pirogov Russian National Research Medical University, Moscow, Russia; ^3^S. Fyodorov Eye Microsurgery Federal State Institution, Moscow, Russia

**Keywords:** optical coherence tomography angiography, epilepsy, antiseizure medications, retinal capillaries, major depressive disorder

## Abstract

**Purpose:**

This study aims to compare optical coherence tomography angiography (OCTA) data in individuals with focal epilepsy and healthy individuals and to investigate the effect of antiseizure medications (ASM) on OCTA data.

**Methods:**

We examined 48 consecutive patients with focal epilepsy and 46 healthy controls. Area and skeleton density of superficial and deep capillary plexuses in the macular area and peripapillary radial capillary plexus were measured.

**Results:**

In general, no differences in OCTA parameters were found between groups of individuals with epilepsy and healthy individuals. A comparison of individuals with epilepsy with and without comorbid major depressive disorder revealed no differences in OCTA data. However, the area and skeleton densities of the perfused capillary retinal vascular bed in the macular region showed a negative association with the use of valproates and modifiers of the presynaptic release machinery, whereas only the skeleton density of the deep capillary plexus showed a positive association with the use of modulators of voltage-gated sodium channels.

**Conclusion:**

OCTA revealed different effects of various ASM groups on the perfused macular capillary bed. These findings suggest that OCTA parameters could serve as potential biomarkers for assessing ASM effects on small vessels and capillaries in the brain.

## Introduction

Structural optical coherence tomography (OCT) is a valuable tool in neurodegenerative disorders. Thinning of the ganglion cell-inner plexiform layer, peripapillary retinal nerve fiber layer, and other retinal structures have been suggested as potential biomarkers for Alzheimer’s and Parkinson’s diseases, multiple sclerosis, and other conditions (1–3). In people with epilepsy (PWE), similar retinal changes detected by structural OCT have also been proposed as a means to detect signs of neurodegeneration and study the effects of drug treatment, among other applications (4, 5).

Approximately 10 years ago, OCT angiography (OCTA) was introduced into clinical practice (6). In ophthalmology, it is mainly used to detect characteristic pathological changes in the ocular vasculature, such as neovascular membranes or non-perfused areas (7). Quantitative OCTA indices are used not only in ophthalmology but also in neurological disorders to measure the impact of disease on the capillary vascular bed (8, 9). However, there are only three reports in the literature on OCTA in PWE, all of which focus on children (10–12). These studies examine OCTA changes in treatment-naïve patients (10) as well as in patients on different antiseizure medications (ASMs).

This study aimed to compare OCTA data in individuals with focal epilepsy and healthy individuals and to investigate the effect of ASM on OCTA.

## Materials and methods

### Subjects

In this observational cross-sectional study, we included 48 consecutive patients with focal epilepsy admitted to the Moscow Research and Clinical Center for Neuropsychiatry between October 2020 and August 2021 (Epilepsy group). The inclusion criteria were as follows: individuals aged 18 years or older, with a diagnosis of focal epilepsy, confirmed through consensus of at least two experienced epileptologists, in accordance with the International League against Epilepsy (ILAE) classification of epilepsy (13). The exclusion criteria were as follows: smoking; severe psychiatric comorbidities (schizophrenia, psychosis, intellectual disability, or dementia), other than major depressive disorder (MDD); a history of psychogenic non-epileptic seizures; diabetes mellitus; severe somatic, neurological, or ophthalmic disorders (including high myopia >6.0 diopters and previous ocular surgery); and active COVID-19 (ICD U 07.1, U 07.2), based upon a positive COVID-19 polymerase chain reaction (PCR) test.

A total of 24 (50%) of the 48 patients in our cohort had structural focal epilepsy (due to traumatic brain injury, stroke, malformations of cortical development, etc.), while the etiology of the remaining cases of focal epilepsy was unknown. The duration of epilepsy was 14.7 ± 11.9 years, and the age of epilepsy onset was 22.4 ± 16.0 years. Only 14 (29%) of the 48 PWE were seizure-free. Twelve PWE had one to two seizures per year, 10 PWE had less than one seizure per month, and 12 PWE had more than one seizure monthly. Twenty (42%) PWE were drug-resistant.

PWE were not treatment-naïve and had mostly received ASMs from the following groups for ≥14 days before screening: modulators of voltage-gated sodium channels (carbamazepine, lacosamide, lamotrigine, and oxcarbazepine), modifiers of the presynaptic release machinery (pregabalin, gabapentin, levetiracetam, brivaracetam, and ethosuximide), and valproates (valproic acid and sodium valproate) (14).

The diagnosis of comorbid MDD [current depressive episode, (cDE)] was made by a psychiatrist experienced in mood disorders, based on the ICD-10 criteria.

Forty-six age- and sex-matched healthy volunteers formed the control group.

This study adhered to the tenets of the Declaration of Helsinki and received approval from the local ethics committee. Informed consent was obtained from all subjects before participation.

### OCTA

OCTA was performed with the OCT HS-100 (Canon Inc., Tokyo, Japan) using Angio eXpert, OCTA version 2.0 (Tokyo, Japan). The OCT HS-100 is a spectral-domain OCT with a scanning rate of 70.000 A-scans/s and a central wavelength of 855 nm. Its declared axial resolution in tissue is 3 μm, and lateral resolution is 20 μm. Structural OCT was also performed to exclude concomitant ophthalmic disorders.

In all subjects, only the right eye was examined without pupil dilation (the choice between the right and left eyes was made randomly). Scans with signal strength ≥7 were included. To measure quantitative OCTA parameters, two protocols were used. The “OCTA (6 × 6 mm) (Macula)” protocol measures the superficial and deep capillary plexuses in the macular area. This area is divided according to the Early Treatment Diabetic Retinopathy Study (ETDRS) scheme into a central (1 mm diameter) and four parafoveal zones, forming an inner ring (annulus) with an inner diameter of 1 mm and an outer diameter of 3 mm. In the central zone, the foveal avascular zone (FAZ) is measured semi-automatically. The “OCTA (4 × 4 mm) (Disc)” protocol measures the peripapillary radial capillary plexus, which is divided into four peripapillary zones, forming an annulus with a size of 1–3 mm.

All OCTA protocols provide two types of measurements—area density and skeleton density. Area density is “the percentage of white pixels in the region (%).” Skeleton density is defined as follows: “This function transforms the lines of a binary image created from an OCTA image into thin lines and indicates the value obtained by dividing the sum of the length of the thin lines in the region by the area (mm^−1^)” [OCT HS-100 Operation Manual. Canon Inc., 2019, p.130]. Area density reflects the area of active (perfused) vessels forming the vascular (mostly capillary) bed in a given region. Unlike area density, skeleton density does not depend on the vessels’ size (diameter) and reflects only the number and total length of the vessels. It should be noted that OCTA terminology is not standardized. In other OCTA devices, area density is also referred to as perfusion density, vessel density, or vessel area density. Skeleton density is also referred to as vessel density or skeleton area density, or it may not be measured at all.

To simplify the comparison of area and skeleton density across different groups of patients, this study calculated the average densities in four parafoveal or four peripapillary zones (quadrants).

### Statistical analysis

Statistical analysis was performed using the R software package, version 4.1.2 (The R Foundation for Statistical Computing, http://www.R-project.org, accessed 11 March 2022) and Jamovi software, version 2.3.28 (Jamovi project, https://www.jamovi.org, accessed 06 August 2024). The normality of distributions was assessed using the Kolmogorov–Smirnov and Shapiro–Wilk tests. Normally distributed variables are presented as mean (M) ± standard deviation (SD), whereas non-normally distributed variables are presented as median (Me) and interquartile range (IQR). A comparison of continuous variables in two groups was performed using Student’s *t*-test for independent samples, Welch’s *t*-test, or Mann–Whitney *U*-test, as appropriate. Categorical variables were compared using Fisher’s exact test. Multivariate linear regression analysis was performed to assess the association between OCTA parameters and demographic parameters, epilepsy duration, seizure frequency, ASM influence, etc. *p <* 0.05 was considered statistically significant.

## Results

### Possible MDD influence on OCTA data in individuals with focal epilepsy

A psychiatric evaluation found MDD (cDE) in 25 out of 48 PWE. A comparison of groups of patients with focal epilepsy and concomitant MDD (group MDD (+), *n* = 25) and with focal epilepsy without MDD (group MDD (−), *n* = 23) did not show differences in age and sex (Table 1) and did not reveal any differences in OCTA parameters (averages of four quadrants) (Table 2). This corresponds to the results obtained in our previous study (15) and allowed further analysis without taking into account the presence of concomitant MDD.

**Table 1:** Age and sex of focal epilepsy patients with concomitant MDD—group MDD (+) and without MDD—group MDD (−).

Group	MDD (+) *n* = 25	MDD (−) *n* = 23	*p*
Parameter			
Age, years, mean ± standard deviation (range)	36.8 ± 10.8 (20–56)	37.5 ± 13.2 (19–69)	0.836^a^
Sex, F/M, *n*	20/5	14/9	0.207^b^

MDD, major depressive disorder; ^a^Student’s *t*-test; ^b^Fisher’s exact test.

**Table 2:** Optical coherence tomography angiography parameters in focal epilepsy patients with concomitant MDD—group MDD (+) and without MDD—group MDD (−), median (interquartile range).

Group	MDD (+) *n* = 25	MDD (−) *n* = 23	*p^a^*
Parameter (averages of four quadrants)			
Superficial capillary plexus: area density (%)	38.0* (37.2–38.5)	37.5* (36.2–38.4)	0.198
Superficial capillary plexus: skeleton density (mm/mm^2^)	23.7* (22.6–24.4)	23.6* (19.5–24.6)	0.590
Deep capillary plexus: area density (%)	39.5* (38.0–41.5)	36.9 (26.4–41.0)	0.069
Deep capillary plexus: skeleton density (mm/mm^2^)	27.4* (25.8–29.2)	26.9 (16.9–29.6)	0.489
Peripapillary radial capillary plexus: Area density (%)	42.1* (40.3–43.3)	42.5* (40.9–44.3)	0.509
Peripapillary radial capillary plexus: Skeleton density, mm/mm^2^	26.3* (23.9–27.2)	26.8* (24.1–27.8)	0.609

MDD, major depressive disorder; ^a^Mann–Whitney *U*-test; *one patient excluded due to incomplete data.

### OCTA in individuals with focal epilepsy vs. controls

To study the influence of focal epilepsy on OCTA parameters the comparison was performed between the epilepsy and control groups. Age and sex did not differ in these groups (Table 3). No differences were found in the averaged four quadrants area and skeleton densities (Table 4) and in all other OCTA parameters (Table 5).

**Table 3:** Age and sex of patients in the epilepsy and control groups.

Group	Epilepsy *n* = 48	Control *n* = 46	*p*
Parameter			
Age, years, mean ± SD (range)	37.2 ± 11.9 (19–69)	33.2 ± 10.3 (22–63)	0.093 ^a^
Sex, Female/Male	34/14	37/9	0.341 ^b^

SD, standard deviation; ^a^Student’s *t*-test; ^b^Fisher’s exact test.

**Table 4:** Optical coherence tomography angiography parameters in the epilepsy and control groups, median (interquartile range).

Group	Epilepsy *n* = 48	Control *n* = 46	*p*
Parameter (averages of four quadrants)			
Superficial capillary plexus: area density (%)	37.9** (37.0–38.5)	37.9* (37.3–38.7)	0.321 ^a^
Superficial capillary plexus: skeleton density (mm/mm^2^)	23.7** (20.2–24.4)	23.6* (22.5–24.4)	0.640 ^a^
Deep capillary plexus: area density (%)	39.1* (30.2–41.5)	39.6** (37.1–41.0)	0.502 ^a^
Deep capillary plexus: skeleton density (mm/mm^2^)	27.1* (20.6–29.5)	27.8** (24.2–29.0)	0.868 ^a^
Peripapillary radial capillary plexus: Area density (%) mean ± SD (range)	41.8 ± 2.9** (32.5–46.5)	42.0 ± 1.8* (36.5–44.5)	0.630 ^b^
Peripapillary radial capillary plexus: skeleton density, mm/mm^2^	26.5** (23.8–27.8)	26.9* (26.0–27.2)	0.518 ^a^

SD, standard deviation; ^a^Mann–Whitney *U*-test; ^b^Welch’s *t*-test (Levene’s test *p* < 0.05); *one subject excluded due to incomplete data; **two subjects excluded due to incomplete data. Differences with the control group are not significant.

**Table 5:** Optical coherence tomography angiography parameters in the epilepsy and control groups, mean ± standard deviation (range).

Group	Epilepsy *n* = 48	Control *n* = 46	*p*
Parameter			
Foveal avascular zone (mm^2^)*			
Superficial capillary plexus	0.26 ± 0.11 (0.06–0.56)	0.26 ± 0.11 (0.03–0.61)	0.963 ^a^
Deep capillary plexus	0.39 ± 0.11 (0.11–0.58)	0.38 ± 0.13 (0.13–0.64)	0.677 ^a^
Superficial capillary plexus: Area density (%)**			
Central subfield	28.57 ± 4.82 (14.5–41.6)	27.48 ± 3.77 (18.3–33.9)	0.226 ^a^
Superior quadrant Me (IQR)	38.0 (36.7–38.8)	38.4 (37.6–39.4)	0.103 ^c^
Nasal quadrant Me (IQR)	37.3 (36.2–38.5)	37.3 (36.6–38.5)	0.598 ^c^
Inferior quadrant	38.19 ± 3.16 (22.9–42.8)	38.89 ± 1.40 (35.8–42.1)	0.171 ^b^
Temporal quadrant Me (IQR)	37.2 (36.0–37.9)	37.2 (36.2–38.2)	0.479 ^c^
Average of four quadrants Me (IQR)	37.9 (37.0–38.5)	37.9 (37.3–38.7)	0.321 ^c^
Superficial capillary plexus: Skeleton density (mm/mm^2^)**			
Central subfield	18.3 ± 4.8 (7.1–37.5)	17.4 ± 3.0 (10.2–22.8)	0.289 ^a^
Superior quadrant, Me (IQR)	23.0 (19.9–24.1)	23.5 (22.4–24.4)	0.124 ^c^
Nasal quadrant, Me (IQR)	24.0 (21.5–24.8)	23.7 (22.9–24.7)	0.975 ^c^
Inferior quadrant	23.6 ± 4.0 (15.0–40.9)	23.7 ± 1.8 (19.2–26.7)	0.818 ^b^
Temporal quadrant Me (IQR)	23.5 (20.5–24.3)	23.2 (21.9–24.1)	0.891 ^c^
Average of four quadrants Me (IQR)	23.7 (20.2–24.4)	23.6 (22.5–24.4)	0.640 ^c^
Deep capillary plexus: Area density (%), Me (IQR)***			
Central subfield	33.8 ± 8.1 (7.6–46.6)	35.8 ± 5.7 (17.9–43.2)	0.181 ^a^
Superior quadrant	33.8 ± 9.2 (1.2–43.1)	35.8 ± 6.5 (18.4–44.7)	0.226 ^a^
Nasal quadrant	37.6 ± 7.1 (21.6–44.9)	39.4 ± 5.2 (21.7–45.2)	0.179 ^b^
Inferior quadrant, Me (IQR)	39.3 (31.8–42.0)	40.0 (37.7–42.2)	0.246 ^c^
Temporal quadrant	36.0 ± 6.8 (16.5–44.4)	38.3 ± 3.9 (25.8–43.0)	0.051 ^b^
Average of four quadrants Me (IQR)	39.1 (30.2–41.5)	39.6 (37.1–41.0)	0.502 ^c^
Deep capillary plexus: Skeleton density (mm/mm^2^)***			
Central subfield	24.0 ± 7.1 (4.5–38.4)	25.2 ± 5.1 (10.9–33.3)	0.367 ^a^
Superior quadrant	23.4 ± 7.4 (0.6–32.1)	24.4 ± 5.3 (11.3–31.2)	0.449 ^a^
Nasal quadrant, Me (IQR)	28.3 (21.0–30.9)	28.4 (25.0–30.2)	0.915 ^c^
Inferior quadrant, Me (IQR)	28.4 (19.4–29.5)	28.4 (25.4–29.5)	0.515 ^c^
Temporal quadrant, Me (IQR)	27.0 (21.8–28.9)	27.8 (24.1–29.1)	0.355 ^c^
Average of four quadrants Me (IQR)	27.1 (20.6–29.5)	27.8 (24.2–29.0)	0.868 ^c^
Peripapillary radial capillary plexus: Area density (%)**			
Superior quadrant	42.3 ± 4.1 (32.7–50.1)	42.6 ± 4.4 (31.6–52.5)	0.677 ^a^
Nasal quadrant	39.6 ± 4.8 (24.9–45.4)	39.3 ± 3.3 (32.0–45.8)	0.728 ^b^
Inferior quadrant	42.7 ± 5.1 (29.8–51.9)	43.5 ± 3.7 (32.1–50.0)	0.405 ^b^
Temporal quadrant	42.6 ± 2.6 (32.6–45.8)	43.1 ± 2.0 (36.8–47.8)	0.368 ^a^
Average of four quadrants	41.8 ± 2.9 (32.5–46.5)	42.0 ± 1.8 (36.5–44.5)	0.630 ^b^
Peripapillary radial capillary plexus: Skeleton density (mm/mm^2^)**			
Superior quadrant	24.3 ± 4.2 (15.1–31.9)	25.0 ± 4.6 (13.0–39.4)	0.454 ^a^
Nasal quadrant	23.2 ± 4.4 (14.7–30.0)	22.8 ± 3.7 (15.8–29.7)	0.704 ^a^
Inferior quadrant	25.5 ± 4.3 (14.6–32.1)	26.3 ± 3.6 (15.1–32.2)	0.349 ^a^

Temporal quadrant	29.0 ± 3.2 (18.5–33.0)	29.9 ± 3.3 (19.5–34.8)	0.191 ^a^
Average of four quadrants Me (IQR)	26.5 (23.8–27.8)	26.9 (26.0–27.2)	0.518 ^c^

Me, median; IQR, interquartile range. ^a^Student’s *t*-test, ^b^Welch’s *t*-test (Levene’s test *p* < 0.05), ^c^Mann–Whitney *U*-test. *Two patients excluded due to incomplete data. **Two patients and one control excluded due to incomplete data. ***One patient and two controls excluded due to incomplete data Differences with the control group are not significant.

### Influence of antiseizure medications and other factors on OCTA

Taking into account that more than 60% of PWE received antiseizure medications (ASM) in two to three groups, an analysis of the influence of ASMs and other factors on OCTA in PWE was performed using multivariate linear regression. Demographic parameters (age, sex, education, etc.) and epilepsy features (seizure frequency, epilepsy duration, etc.) did not show any significant impact on OCTA. The ASMs of two groups (valproates and modifiers of the presynaptic release machinery) showed a significant negative effect on the area and skeleton densities of both superficial and deep capillary plexuses in the macular region. In contrast, only the skeleton density of the deep capillary plexus showed a positive association with the use of modulators of voltage-gated sodium channels (Table 6). No similar effects were found for the peripapillary radial capillary plexus.

**Table 6:** Influence of antiseizure medications (ASM) on optical coherence tomography angiography parameters: multivariate linear regression results.

Parameter	Group of ASMs	multiple R^2^	P	β
(averages of four quadrants)				
Superficial capillary plexus: Area density (%)	Valproates	0.156	0.026	−0.295
	Modifiers of the presynaptic release machinery			−0.233
Superficial capillary plexus: Skeleton density (mm/mm^2^)	Valproates	0.187	0.012	−0.297
	Modifiers of the presynaptic release machinery			−0.284
Deep capillary plexus: Area density (%)	Valproates	0.256	0.002	−0.439
	Modifiers of the presynaptic release machinery			−0.197
Deep capillary plexus: Skeleton density (mm/mm^2^)	Modulators of voltage-gated sodium channels	0.188	0.010	0.273
	Valproates			−0.243

β, standardized estimates.

**Figure 1:**
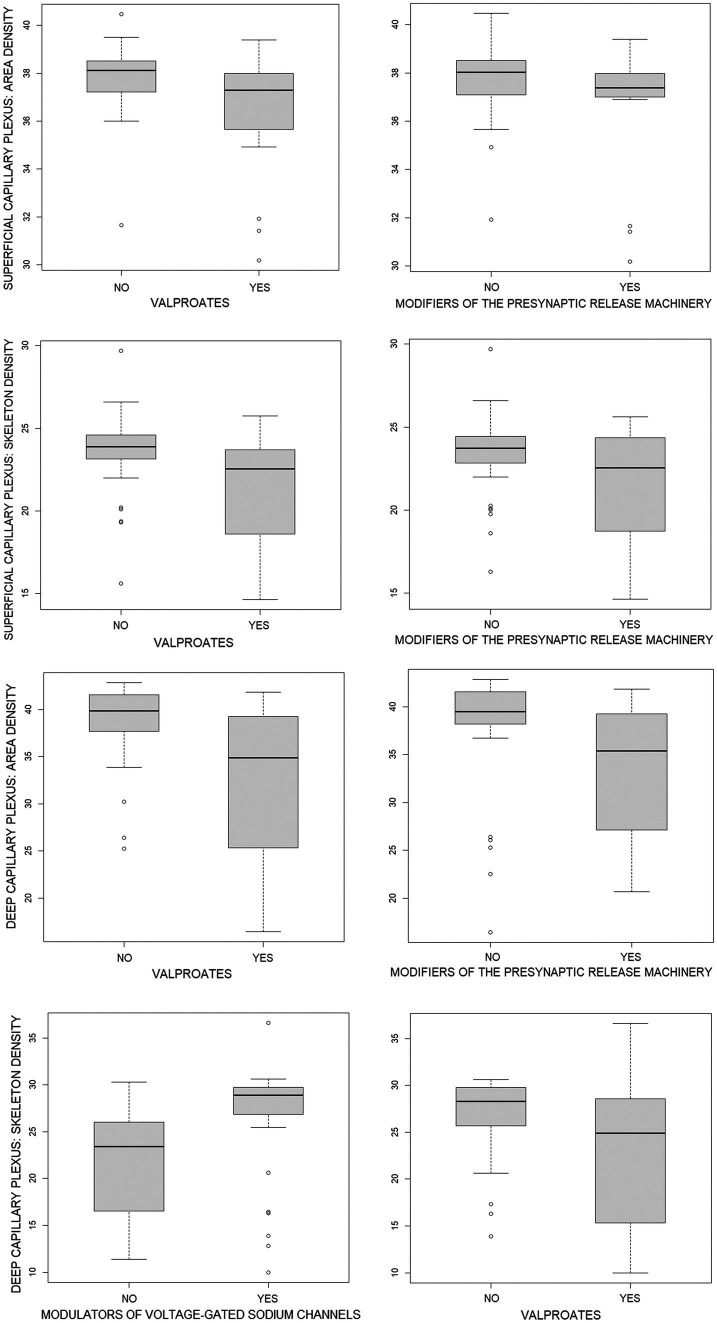
Boxplots of optical coherence tomography angiography parameters in patients who did (YES) and did not (NO) receive a particular group of antiseizure medications (ASMs) as presented in Table 6. Quantitative data are given in Table 7. The position of boxplots of each ASM corresponds to the ASM position in Tables 6, 7.

The associations reported in Table 6 are illustrated in Figure 1 and Table 7, which provide a direct comparison of OCTA parameters in patients who received versus those who did not receive a particular group of ASMs, as presented in Table 6. It should be noted that there were no age differences between the compared groups, while groups of patients who received and did not receive modulators of voltage-gated sodium channels differed significantly in terms of sex. However, multiple regression revealed a very weak influence of sex and age on the OCTA parameters studied.

**Table 7:** Optical coherence tomography angiography parameters in patients who did and did not receive a particular group of antiseizure medications (ASM), median (interquartile range).

Parameter	ASM group	ASM (+)	ASM (−)	*p^a^*
(averages of four quadrants)				
Superficial capillary plexus: Area density (%)	Valproates n1 = 19; n0 = 28	37.3* (35.8–38.0)	38.1 (37.3–38.5)	NS
	Modifiers of the presynaptic release machinery n1 = 16; n0 = 31	37.4* (37.0–38.0)	38.0 (37.1–38.5)	NS
Superficial capillary plexus: Skeleton density (mm/mm^2^)	Valproates n1 = 19; n0 = 28	22.6* (18.9–23.7)	23.9 (23.2–24.6)	0.033
	Modifiers of the presynaptic release machinery n1 = 16; n0 = 31	22.6* (18.7–24.4)	23.7 (22.8–24.4)	NS
Deep capillary plexus: Area density (%)	Valproates n1 = 19; n0 = 28	34.9 (25.3–39.3)	39.8 (37.9–41.5)	0.005
	Modifiers of the presynaptic release machinery n1 = 16; n0 = 31	35.4 (28.0–39.2)	39.5 (38.2–41.6)	0.021
Deep capillary plexus: Skeleton density (mm/mm^2^)	Modulators of voltage-gated sodium channels n1 = 33; n0 = 14	28.9 (26.9–29.8)	23.4 (16.7–25.7)	0.005
	Valproates n1 = 19; n0 = 28	24.9 (15.3–28.6)	28.3 (25.8–29.8)	0.026

ASM (+), ASM (−)—groups of patients who did and did not receive ASM; n1, n0—numbers of patients in these groups. ^a^ – Mann–Whitney U-test. *One person excluded due to incomplete data. NS—non-significant.

Figure 1 and Table 7 show that the capillary retinal vascular bed is significantly decreased in patients receiving valproates or modifiers of the presynaptic release machinery while the skeleton density of the deep capillary plexus in patients on modulators of voltage-gated sodium channels is significantly increased.

## Discussion

In our study, we examined patients with focal epilepsy who were primarily taking ASMs from three groups: valproates, modifiers of the presynaptic release machinery, and modulators of voltage-gated sodium channels. For the first time, a negative association was found between the area and skeleton densities of both superficial and deep macular capillary plexuses and the use of valproates or modifiers of the presynaptic release machinery, indicating a decrease in the perfused retinal capillary bed. In contrast, the modulators of voltage-gated sodium channels demonstrated a positive association with the skeleton density of the deep capillary plexus, equivalent to an increase in the deeper portion of the perfused retinal capillary bed. When ASMs were not taken into account, OCTA did not show any differences between patients and controls. It could be due to an approximately equal number of prescribed ASMs causing an increase and decrease in the perfused retinal capillary bed.

OCTA is a relatively new technique. There are only a few publications on its use in patients with epilepsy (10–12). All of these studies were conducted in children and included mixed groups of patients with focal and generalized epilepsies. One article compared untreated patients with a control group (12). Another compared children treated with levetiracetam with a control group (11), and a third compared three groups of epilepsy patients taking three different ASMs (carbamazepine, levetiracetam, and valproic acid) with a control group (10). All these studies did not find differences in any indices in superficial and deep capillary macular plexuses as well as in peripapillary radial capillary plexus. Moreover, all three papers found differences in choroidal perfusion, which, compared to controls, was decreased in untreated patients (12) and in patients on valproic acid (10). It was also shown to be decreased in children taking levetiracetam (11); however, this conclusion was not confirmed in another study (10).

With choroidal perfusion decrease in untreated patients (12), it is not clear which part of the decrease in treated persons is due to epilepsy and which is due to ASMs. It should be also noted that unlike superficial and deep capillary macular plexuses or peripapillary radial capillary plexus, which are measured automatically by software incorporated in the OCT equipment, choroidal perfusion was measured on exported images using analysis by the ImageJ program (National Institutes of Health, Bethesda, MD, USA). This analysis is not automatic and therefore may produce much more subjective results.

The retina is closely connected to the brain, both structurally and developmentally, and is often considered an extension of it. This relationship is also reflected in the similarities of the vascular system at the micro level, as well as in their comparable vascular reactions. At present, there are no studies on possible associations between OCTA parameters and cerebral blood flow in epilepsy. However, in other diseases, there are examples of strong connections between the changes in OCTA and structural and functional changes in the vascular system of the brain. For example, lower vessel density (aka area density in our study) on OCTA was associated with lower cerebrovascular reactivity to CO2 and higher mean diffusivity on diffusion tensor imaging, reflecting subvisible white matter damage in patients with cerebral small vessel disease (16). Lower vessel density of superficial capillary plexus in this category of patients was associated with white matter hyperintensity scores (17). The results of these and similar studies (18) suggest that OCTA parameters could serve as potential biomarkers of cerebral small vessel disease (16–19).

Changes in vascular density (aka skeleton density in our study) of the superficial capillary plexus, correlated with brain perfusion parameters (cerebral blood flow, cerebral blood volume, etc.) in patients with moderate or severe internal carotid artery stenosis (20). In addition to OCTA, other retinal vascular parameters, such as arteriovenous ratio, were correlated with cerebral blood flow, which was shown in patients with bipolar disorder (21).

Based on these data, it can be expected that the effects of ASM on OCTA parameters identified in the present study may be accompanied by similar effects on cerebral capillaries.

There are studies of the influence of ASMs on cerebral blood flow in PWE (22, 23). In one of them, valproate diminished global cerebral blood flow in healthy volunteers, which was consistent with our OCTA findings (22). In another study, lamotrigine was found to reduce perfusion in cortico-thalamo-limbic areas, the orbitofrontal cortex, and the brainstem in patients with drug-naïve idiopathic generalized epilepsy (23). The direction of these changes is not consistent with our OCTA data. However, the opposite changes were observed only in deep capillary plexus skeleton density, and the patients in these studies were quite different. Both of these studies involve expensive, high-tech techniques such as positron emission tomography (PET) (22) or single-photon emission computed tomography (SPECT) (23). If OCTA provides comparable information, it could have an important place in ASM research. This will be the subject of our future studies.

### Limitations

The comparison groups of patients who received and did not receive a particular group of ASMs are relatively small, and some patients received ASMs from two or more groups. The reported effects of ASMs on OCTA need to be confirmed in larger groups of patients receiving ASM monotherapy.

The relationship between the capillary vascular bed of the retina and the brain, as suggested by existing literature, was not explored in this study. This topic requires a separate investigation using special methods to assess the condition of small cerebral vessels and cerebral blood flow.

## Conclusion

OCTA revealed different effects across various ASM groups on the perfused macular capillary bed. These findings suggest that OCTA parameters could serve as potential biomarkers for assessing ASM effects on small vessels and capillaries in the brain.

## Data Availability

The raw data supporting the conclusions of this article will be made available by the authors, without undue reservation.
